# Estimating the cost and cost‐effectiveness of adding zinc to, and improving the performance of, Burkina Faso's mandatory wheat flour fortification programme

**DOI:** 10.1111/mcn.13515

**Published:** 2023-04-06

**Authors:** Katherine P. Adams, Michael Jarvis, Stephen A. Vosti, Mari S. Manger, Ann Tarini, Jérome W. Somé, Hervé Somda, Christine M. McDonald

**Affiliations:** ^1^ Institute for Global Nutrition University of California Davis California USA; ^2^ Independent Consultant Washington DC District of Columbia USA; ^3^ Department of Agricultural and Resource Economics University of California Davis California USA; ^4^ International Zinc Nutrition Consultative Group University of California San Francisco California USA; ^5^ Independent Consultant Laval Quebec Canada; ^6^ IZiNCG Fortification Task Force San Francisco California USA; ^7^ Institut de Recherche en Sciences de la Santé Centre National de Recherche Scientifique et Technologique Ouagadougou Burkina Faso; ^8^ Développement Agricole et Transformation de l'Agriculture University of Thomas Sankara Ouagadougou Burkina Faso; ^9^ Department of Pediatrics, School of Medicine University of California San Francisco California USA

**Keywords:** Burkina Faso, cost, cost‐effectiveness, large‐scale food fortification, nutrition policy, wheat flour fortification, zinc

## Abstract

Zinc is an essential micronutrient that promotes normal growth, development and immune function. In the context of persistent dietary zinc inadequacies, large‐scale food fortification can help fill the gap between intake and requirements. Burkina Faso mandates wheat flour fortification with iron and folic acid. We used activity‐based cost modelling to estimate the cost of adding zinc to the country's wheat flour fortification standard assuming (1) no change in compliance with the national standard, and (2) a substantial improvement in compliance. We used household food consumption data to model effective coverage, that is, the number of women of reproductive age (WRA) predicted to achieve adequate zinc density (zinc intake/1000 kcal) with the addition of fortification to diets. Without interventions, the prevalence of inadequate dietary zinc density was ~35.5%. With no change in compliance, the annual average incremental cost of adding zinc to fortified wheat flour was $10,347, which would effectively cover <1% of WRA at an incremental cost of ~$0.54/WRA effectively covered. Improving compliance added ~$300,000/year to the cost of the fortification programme without zinc; including zinc added another ~$78,000/year but only reduced inadequate intake among WRA by 3.6% at an incremental cost of ~$0.45/WRA effectively covered. Although the incremental cost of adding zinc to wheat flour is low ($0.01/wheat flour consumer/year), given low levels of wheat flour consumption, zinc fortification of wheat flour alone contributes marginally to, but will not fully close, the dietary zinc gap. Future research should explore potential contributions of zinc to a broader set of delivery vehicles.

## Key messages

- Large‐scale food fortification can be an effective strategy for reducing zinc deficiency.
- Dietary zinc inadequacy is a public health problem in Burkina Faso. We modelled the potential effectiveness and cost‐effectiveness of fortifying wheat flour with zinc to help address this issue.
- Although the incremental cost of adding zinc to wheat flour would be low ($0.01/wheat flour consumer/year), given the severity of inadequacy and low levels of wheat flour consumption, zinc fortification of wheat flour alone contributes marginally to, but will not fully close, the dietary zinc gap.
- A broader set of delivery vehicles is needed to effectively address zinc inadequacy in Burkina Faso.

## 1 INTRODUCTION

Micronutrient deficiencies, or “hidden hunger”, remain a significant global health concern, particularly among subpopulations with relatively high micronutrient requirements, including children under 5 years of age and women of reproductive age (WRA) (Stevens et al., 2022; Victora et al., 2021). Adequate zinc intake is necessary for normal growth, development, and immune function, and zinc deficiency is associated with increased incidence of diarrhoea and respiratory infections among children under 5 years of age (King et al., 2016). The global prevalence of risk for inadequate zinc intake has been estimated at 17.3% (Wessells & Brown, 2012), and among 25 low‐ and middle‐income countries (LMICs) with plasma/serum zinc concentration data, the prevalence of zinc deficiency is >20% for at least one physiological group in all but two countries (Gupta et al., 2020). In Burkina Faso, an estimated 35.2% of the population is at risk of inadequate zinc intake (K. R. Wessells, personal communication, December 1, 2022), and a study in rural Burkina Faso found low serum zinc concentrations among 39.4% of women and 63.7% of children 36–59 months of age (Martin‐Prevel et al., 2016).

Alongside interventions to promote dietary diversification and modification, biofortification, and supplementation, large‐scale food fortification (LSFF) is one of the primary intervention strategies identified by the World Health Organization (WHO) and the United Nations Food and Agriculture Organization (FAO) to address micronutrient malnutrition (Osendarp et al., 2018). LSFF, which is the addition of vitamins and/or minerals to staple foods or condiments during industrial processing (Olson et al., 2021; World Health Organization & Food and Agricultural Organization, 2006), does not require changes in dietary patterns to be effective and is often touted as relatively low‐cost. As such, LSFF is widely recognized as a potentially cost‐effective intervention to improve micronutrient intake and reduce the prevalence of deficiency (Horton et al., 2008; Keats et al., 2021; Osendarp et al., 2018; World Health Organization & Food and Agricultural Organization, 2006).

In the past two decades, LMICs have increasingly utilized the fortification of staple foods, including wheat flour, refined oils and rice, as a strategy to improve the micronutrient adequacy of diets (Osendarp et al., 2018). A recent systematic review and meta‐analysis found that zinc fortification with or without other micronutrients reduced the prevalence of zinc deficiency and may provide health and functional health benefits, such as lowering the incidence of child diarrhoea (Tsang et al., 2021). Based on this evidence, the recently updated 2022 WHO wheat flour fortification guidelines include the conditional recommendation to fortify wheat flour with zinc as a public health strategy to improve serum/plasma zinc status (World Health Organization, 2022). However, of the 40 LMICs for which zinc deficiency is a public health problem, only 17 include zinc in their mandatory fortification standards (Global Fortification Data Exchange, 2022; Tarini et al., 2021; K. R. Wessells, personal communication, December 1, 2022).

Sorghum, millet, and maize are the main staple grains consumed in Burkina Faso (FEWS NET, 2017). Of the cereal grains that are most commonly utilized for LSFF (wheat flour, maize flour and rice), in Burkina Faso wheat flour is the most feasible fortifiable food vehicle, as little to no maize flour or rice in the food system is industrially processed, while all wheat flour in Burkina Faso is industrially processed and, hence, fortifiable (Global Fortification Data Exchange, 2022). In 2012, in accordance with the regional West African Economic and Monetary Union (UEMOA) fortification standard that was subsequently made mandatory in all eight member countries, Burkina Faso implemented legislation mandating the fortification of domestically processed and imported wheat flour with iron and folic acid (Global Fortification Data Exchange, 2022; Grant et al., 2018). However, despite the estimated high burden of zinc deficiency in the country, zinc was not included in the fortification standard because, at the time the regional standard was developed, there was considered to be a lack of evidence of the efficacy and effectiveness of zinc fortification (Tarini et al., 2021). As such, and because wheat flour is already required to be fortified with iron and folic acid, revising the wheat flour fortification standard in Burkina Faso to also include zinc may be a relatively straightforward and low‐cost strategy to increase dietary zinc intakes.

The success of a LSFF programme in terms of its ability to cost‐effectively improve micronutrient intake and status depends on appropriate programme design (or redesign) and effective implementation and management (Adams, Vosti, et al., 2022; Mkambula et al., 2020). This includes determining whether industrially processed forms of the candidate food vehicle are regularly consumed in sufficient quantities, such that fortification would help close the gap between dietary intake and requirements by population subgroups at greatest risk for deficiency. It also includes understanding the range of costs associated with implementing and managing an LSFF programme as well as planning for how different components of the cost will be paid (e.g., paid for or subsidized by government, borne by industry, passed on to consumers, etc.). Including cost as fundamental input into the overall evidence base for decision‐making can help prevent situations in which an intervention with great potential to help address micronutrient deficiencies is either never implemented or underperforms due to high and/or unexpected costs. Given the well‐documented challenges of achieving the full potential of LSFF due, in part, to weak monitoring and enforcement systems (Luthringer et al., 2015), understanding the investments that must be made to strengthen and sustain these systems is also a critical component of this evidence base (Heidkamp et al., 2021).

The Micronutrient Intervention Modelling (MINIMOD) project has developed a set of modelling tools to provide policymakers with estimates of the nutritional benefits, costs, and cost‐effectiveness of alternative micronutrient intervention programmes (Adams, Luo, et al., 2022; Engle‐Stone, Nankap, et al., 2015; Vosti et al., 2015). Our objectives were to apply the MINIMOD modelling framework and its underlying data to estimate the incremental nutritional benefits, costs and cost‐effectiveness of adding zinc to Burkina Faso's existing wheat flour fortification programme. As described below, Burkina Faso's wheat flour fortification programme is underperforming relative to the goals set out in the country's national wheat flour fortification standard. Given this, we also estimated the costs associated with improving compliance with the national standard and the incremental cost and cost‐effectiveness of adding zinc to the wheat flour fortification standard under this improved compliance scenario.

## 2 METHODS

### 2.1 Cost modelling scenarios

We estimated the incremental cost of adding zinc (in the form of zinc oxide) to Burkina Faso's wheat flour fortification programme. More specifically, we modelled the costs of moving from the status quo of wheat flour fortified with only iron and folic acid to the addition of zinc included in the country's wheat flour fortification standard. Our analysis considered two levels of programme compliance vis‐à‐vis the standard (current compliance and improved compliance); in all, we modelled four distinct wheat flour fortification scenarios (Table 1). The costs associated with the *current programme* scenario were based on Burkina Faso's current wheat flour fortification standard, where assumptions about compliance with the national standard (which influence both programme nutritional benefits and costs) were based on the 2018 Fortification Assessment Coverage Toolkit (FACT) survey data collected by the Global Alliance for Improved Nutrition that measured the iron content of wheat flour samples collected at markets across Burkina Faso (Global Alliance for Improved Nutrition, 2018). Note that we assumed no degradation of iron fortificant from the point of fortification to markets (Hemery et al., 2018). The *expanded current programme* scenario incorporated the hypothetical addition of zinc to the national wheat flour fortification standard, while maintaining assumptions about compliance with the national standard as in the current programme scenario. The *improved compliance* scenario included the estimated costs of improving the compliance of the current programme (i.e., fortification of wheat flour with iron and folic acid) along two dimensions: (1) the percentage of fortifiable wheat flour that is fortified to any extent, and (2) among fortified wheat flour, the average fortification level relative to the national standard. Finally, the *expanded programme with an improved compliance* scenario combined the costs of the expanded current programme (i.e., the hypothetical addition of zinc to the national standard) with the costs associated with improving compliance.

**Table 1:** Wheat flour fortification modelling scenarios.

	Current programme	Expanded current programme	Current programme with improved compliance	Expanded programme with improved compliance
Wheat flour fortification standard	Folic acid (2.5 mg/kg folic acid) and iron (60 mg/kg ferrous fumarate)^a^	Folic acid (2.5 mg/kg folic acid), iron (60 mg/kg ferrous fumarate), and zinc (95 mg/kg zinc oxide)^b^	Folic acid (2.5 mg/kg folic acid) and iron (60 mg/kg ferrous fumarate)^a^	Folic acid (2.5 mg/kg folic acid), iron (60 mg/kg ferrous fumarate), and zinc (95 mg/kg zinc oxide)^b^
Percent of fortifiable^f^ wheat flour that is fortified to any extent	61.5%^c^	61.5%^c^	90%^e^	90%^e^
Among fortified wheat flour, average fortification level as a percent of the national standard	12%^d^	12%^d^	100%^e^	100%^e^

^a^ Source: Global Fortification Data Exchange (2022).

^b^ Source: hypothetical modelling informed by the World Health Organization wheat flour fortification guidelines, assuming average per capita consumption of low‐extraction wheat flour of <75 g/day (World Health Organization, 2022).

^c,d^ Source: Global Alliance for Improved Nutrition (2017).

^e^ Source: hypothetical modelling informed by input from key informants in Burkina Faso collected in September 2022.

^f^ All wheat flour in Burkina Faso is industrially processed and hence assumed to be fortifiable (Global Fortification Data Exchange, 2022).

### 2.2 Costing approach

We took an activity‐based (or microcosting) approach to estimating the economic cost of each of the scenarios outlined above (World Health Organization, 2003), and we defined costs from a societal perspective, that is, including the costs paid by all stakeholders (including industry, the government, nongovernmental organizations, passed on to wheat flour consumers, etc.). Thus, our cost estimates also included costs borne outside Burkina Faso to fortify imported wheat flour, though those costs would likely be passed on to wheat flour consumers in Burkina Faso. We developed four scenario‐specific, activity‐based cost models, with all models incorporating the costs of all of the activities required to plan, implement, operate, and manage the wheat flour fortification programme. To estimate the annual cost of each activity, we identified the quantity of physical inputs required to perform each activity, such as labour, equipment and supplies, shipping and transportation, and so forth, and multiplied these by their respective values. We modelled costs over a 10‐year time horizon (2022–2031) to allow for scenarios that include periods of time during which start‐up costs but no nutritional benefits accrue.

Because the *current programme* scenario reflected the ongoing wheat flour fortification programme as it is currently being implemented in Burkina Faso, the cost model for this scenario was comprised strictly of recurring costs in each year of the 10‐year time horizon. These recurring costs included industry‐related costs to acquire premix, fortify wheat flour, conduct internal quality assurance and quality control(QA/QC) activities at mills, and provide training/retraining to mill personnel involved in fortification and QA/QC activities. Because monitoring and evaluation (M&E) of Burkina Faso's LSFF programme is currently essentially nonexistent, no government‐related recurring costs were included. The *expanded current programme* scenario, which added zinc to the wheat flour fortification standard, included 2 years of “start‐up” costs that accounted for the time and costs associated with planning for and revising the national standard as well as implementing the revised standard (including training for mill personnel and relabelling of wheat flour packaging to reflect the addition of zinc). Recurring costs under *the expanded current programme* scenario were the same as those modelled in the current programme scenario, except that the cost of the micronutrient premix also included zinc after the start‐up period (i.e., in Years 2024–2031).

The *improved compliance* scenario cost model reflected 2 years of “scale‐up” investments required to overhaul and implement the wheat flour fortification monitoring and enforcement strategy as well as additional recurring activities (and costs) necessary to bring about and sustain improvements in compliance. The scale‐up investments included the formal adoption of an M&E plan, the acquisition of fortification equipment (feeders and dosifiers plus installation costs) for mills not currently fortifying (note that mills already fortifying wheat flour with iron and folic acid could continue to use the same equipment to additionally fortify with zinc), and the acquisition of equipment by mills and the government for testing of iron content. The additional recurring activities included systematic monitoring of wheat flour imports at each port of entry, quarterly inspections at domestic wheat flour mills, annual capacity‐building sessions for inspectors, quarterly social marketing and advocacy campaigns, and more intensive management and administration. Improved compliance also included increased outlays for premixes. Finally, the cost model reflecting the *expanded programme with improved compliance* included all of the improved compliance scenario scale‐up and recurring costs and, in addition, included the “start‐up” costs associated with revising the national standard to include zinc as well as relabelling of wheat flour packaging.

### 2.3 Cost data sources

We tapped multiple sources of data to inform the underlying cost model assumptions, as well as unit cost and quantity parameters values. Our primary sources of data to inform assumptions about current QA/QC and monitoring and enforcement activities, as well as QA/QC and monitoring and enforcement activities under the improved compliance scenarios, were based on discussions with key informants and in‐country collaborators in Burkina Faso and Burkina Faso's draft 2023–2027 M&E plan for LSFF (Ministere de la Sante et de L'Hygiene Publique, 2022). The draft M&E plan detailed the activities and frequencies of those activities for ensuring that both imported and domestically milled wheat flour meet the national standard through import, industry, and commercial monitoring. The draft M&E plan also included a 5‐year budget to implement the plan, and we used some of those budget estimates to inform some of the improved compliance scenario costs. Finally, we referred to the World Health Organization's flour fortification monitoring manual to ensure all recommended monitoring activities were included as part of the improved compliance scenario (World Health Organization, 2021).

Premix costs were based on a premix cost calculator developed to estimate an array of options regarding the numbers and amounts of fortificants included in a given premix, and the choice of fortificants and their prices. Within the MINIMOD premix cost calculator, the price of folic acid, ferrous fumarate and zinc oxide were informed by price estimates provided by an international LSFF programme expert in November of 2021. We also included an 18% value‐added tax and a 7% import duty on the cost of imported premix.

Other assumptions and parameter values were informed by published and grey literature, and other online data sources, including the Global Fortification Data Exchange, Fortification Assessment Coverage Tool market survey data, the UNICEF supply catalogue and the Food Fortification Initiative.

### 2.4 Effectiveness modelling

Our metric of effectiveness was effective coverage, defined as the number or percentage of a target population with inadequate micronutrient intake from dietary intake without any intervention to increase micronutrient intakes who achieve adequate intake after the introduction of a micronutrient intervention programme (Adams, Luo, et al., 2022). While children under 5 years of age are commonly the target population for zinc interventions, LSFF alone is unlikely to meet the zinc requirements of young children because of the relatively small quantities of staple foods consumed and fortification levels designed not to exceed the tolerable upper intake level in any population group. Thus, young children in countries with a high prevalence of zinc deficiency are likely to require additional interventions (Zlotkin & Dewey, 2021). Given this, we selected pregnant and nonpregnant women age 15–49 years (hereafter WRA) as our target population. We modelled the potential effectiveness of adding zinc to Burkina Faso's wheat flour fortification standard using household consumption and expenditure survey data collected in 2018/2019 via the Enquête Harmonisée sur les Conditions de Vie des Ménages (EHCVM) survey. To capture seasonality in consumption, the EHCVM survey data were collected in two waves (with different households surveyed in each wave), from October through December of 2018 and April through July of 2019 (The World Bank, 2022). In the food consumption and expenditures section of the survey, a household respondent was asked to recall the quantity of each of the 138 food items consumed by household members during the 7 days preceding the survey.

We used these food consumption data to estimate household daily apparent consumption of each food item, where *apparent* emphasizes assumptions inherent in using household‐level data, including that all food was consumed by the household without food waste or loss and was distributed within the household in proportion to each household member's age‐ and sex‐specific energy requirements. We estimated daily apparent food consumption by adjusting the total quantity of food apparently consumed by the household to reflect the edible portion and yield factor from cooking, where appropriate, and dividing the resulting quantity by 7 days of recall. We identified extreme outliers in reported quantities of food apparently consumed as the food‐ and region‐specific 95th percentile of apparent household consumption per adult male equivalentand replaced outliers above the 95th percentile with the value at the 95th percentile (Adams, Vosti, et al., 2022).

To estimate daily apparent household nutrient intake, we then matched each food item to a food composition table (FCT) entry, or a weighted average of several FCT entries for aggregate or generic food items, from the West African FCT (Food and Agricultural Organization of the United Nations, 2019) or, where appropriate matches from the West African FCT were not available, supplemented with entries from the Nutrition Coordinating Center Nutrient Database for Standard Reference (Schakel et al., 1988) and the Malawian FCT (MAFOODS, 2019). Specific matches were based on input from in‐country collaborators. We then estimated the apparent zinc density of the household diet, that is, daily apparent zinc intake per 1000 kcal, as the ratio of total daily apparent zinc intake divided by total daily energy intake, expressed per 1000 kcal (Vossenaar, Solomons, Monterrosa, et al., 2019). Estimates of household food consumption based on household survey data are particularly prone to measurement error given that they are based on the recall of one household member using a fixed food list and foods consumed away from home are typically inadequately captured (Smith et al., 2014; Food and Agriculture Organization of the United Nations & The World Bank, 2018). To help address measurement error, we estimated the energy‐adjusted zinc densities of household diets rather than apparent zinc intakes of WRA. Note that while using energy‐adjusted zinc density helps account for measurement errors in reported quantities of foods consumed that are reflective of the typical household diet, it does not address many of the limitations inherent in using household survey data to estimate micronutrient adequacy, including potential systematic under/over‐reporting of foods with nutrient contents that differ from the typical household diet (e.g., foods consumed away from home) (National Institutes of Health & National Cancer Institute, 2023). We return to this issue in the discussion section.

We compared the zinc density of the household diet to critical zinc densities for WRA to assess the adequacy of the household diet for meeting the zinc requirements of WRA. We calculated critical zinc densities for WRA as their age‐ and pregnancy‐status‐specific estimated average requirements divided by their age‐ and pregnancy‐status‐specific energy requirements, expressed per 1000 kcal. To account for zinc absorption, we estimated absorbable zinc using published algorithms (IZiNCG, 2019) and then adjusted the absorbed zinc requirements (2.89 mg/day for nonpregnant WRA and 3.59 mg/g for pregnant WRA) and critical zinc densities based on the estimated percentage absorbed zinc in the household samples. Energy requirements were estimated relative to the FAO/WHO human energy requirement estimate of 2900 kcal for a 65 kg adult male age 18–30 years with moderate physical activity (Food and Agricultural Organization of the United Nations & World Health Organization, 2004).

The zinc density of the household diet was classified as sufficient to meet the requirements of WRA if it was above the critical zinc density. Because the pregnancy status of WRA was not collected in the household survey, we assessed zinc adequacy separately for pregnant and nonpregnant women by (1) assuming all WRA in the sample were pregnant and assessing zinc adequacy based on the requirements of pregnant women, (2) assuming all WRA were not pregnant and assessing adequacy based on the requirements of nonpregnant WRA, and (3) estimating overall zinc adequacy among pregnant and nonpregnant WRA by combining these estimates as a weighted average, where weights were based on estimates of the proportion of WRA current pregnant and not pregnant according to the most recent Demographic and Health Survey data (ICF, 2012). Because nutrient density is a metric of dietary quality, adequacy was based on the assumption that WRA were meeting their age‐ and sex‐specific energy requirements (Vossenaar, Solomons, Muslimatun, et al., 2019).

We modelled the contribution of wheat flour fortified with zinc by multiplying daily apparent household consumption of wheat flour (including reported consumption of wheat flour and wheat flour equivalents from products containing wheat flour, such as bread and biscuits) by the average hypothetical zinc fortification levels under the expanded current programme and expanded current programme with improved compliance scenarios (Table 1) and recalculating the nutrient density of the household diet and prevalence of inadequacy. Finally, effective coverage was calculated as the percentage of WRA moving from inadequate zinc density from dietary intake without any intervention to increase zinc intakes to adequate zinc density with the introduction of zinc to fortified wheat flour.

### 2.5 Cost‐effectiveness

We used the estimates of effective coverage to estimate the potential effectiveness of zinc fortification of wheat flour in Burkina Faso, defined as the total number of WRA effectively covered over the 10‐year time horizon. We assumed no changes in consumption of wheat flour (or other foods in the diet) over the 10‐year time horizon. The projected size of the population of WRA in Burkina Faso each year was based on 2019 United Nations World Population Prospects (United Nations et al., 2019). For both the expanded current programme and expanded improved compliance scenarios, we assumed effective coverage was zero in Years 1 and 2, that is, during the period of planning and implementation of the revised national standards. Incremental cost‐effectiveness was then calculated as the incremental cost per WRA effectively covered, or the difference in the 10‐year incremental cost of moving from the current programme to the expanded programme (or, similarly, moving from improved compliance to expanded improved compliance) divided by the total number of WRA effectively covered under each expanded and/or improved programme scenario.

### 2.6 Sensitivity analyses

We conducted sensitivity analyses to assess the influence of several key parameter values on our cost and cost‐effectiveness results. In particular, we reduced and increased the price of zinc oxide by 20%, from $7.00 per kg in the primary analysis to $5.60 and $8.40. We also modelled a 50% increase in the assumed zinc fortification level, from 95 to 142.5 mg/kg; this higher fortification level is similar to countries with wheat fortification standards that include zinc at levels above the WHO recommendations relative to wheat flour consumption (e.g., Ethiopia). We did not assess the sensitivity of our estimates to potential errors in the estimated nutrient composition of diets.

## 3 RESULTS

### 3.1 The wheat flour industry in Burkina Faso

Burkina Faso does not grow any wheat in‐country, but two industrial‐scale wheat flour mills domestically mill approximately 75% of the country's imported wheat supply (Food Fortification Initiative, 2022; Miller Magazine, 2015). The remaining ~25% of wheat flour is imported. All of the wheat flour in the food system is industrially produced and therefore considered fortifiable (Global Fortification Data Exchange, 2022). Currently, Burkina Faso does not have a formal M&E strategy for ensuring wheat flour that is domestically milled or imported is fortified according to the country's national wheat flour fortification standard, or that wheat flour sold in markets and other retail outlets is properly fortified, packaged and labelled. As previously noted, the most recent data available on the extent of fortification of wheat flour (i.e., compliance with the national standard) suggest that, based on wheat flour samples collected at markets and tested for iron content, ~61.5% of all wheat flour is fortified to any extent (the remaining 38.5% is not fortified), and the average fortification level is well below (12% of) the national standard (Global Alliance for Improved Nutrition, 2017; Global Alliance for Improved Nutrition, 2018).

### 3.2 The cost of wheat flour fortification

The estimated annual average cost of the current wheat flour fortification programme (which is functioning at a low level of compliance) in Burkina Faso was $26,601 (Table 2, 2021 US dollars). Expanding the current programme to include zinc raised the annual average cost of wheat flour fortification by $10,347, which included relabelling, the cost of adding zinc to the premix as well as planning for and implementing the revised national standard. This is equal to an additional ~$0.0004 per capita, or an additional ~$0.001 per wheat flour consumer per year. On a cost per metric ton (MT) of fortified wheat flour basis, adding zinc oxide to the premix increased the total cost from ~$0.53 to ~$0.73/MT. The relative contribution of premix to the total cost was ~61% and ~66% under the current and expanded current programme scenarios, respectively (Supporting Information: Table S1).

**Table 2:** Estimated cost of the current wheat flour fortification programme in Burkina Faso and the cost of expanding the programme to include zinc.

	Current programme^a^ (2021 US dollars)	Expanded current programme^b^ (2021 US dollars)	Incremental cost of adding zinc to the current programme (2021 US dollars)
Total 10‐year cost	$266,010	$369,480	$103,470
Annual average cost	$26,601	$36,948	$10,347
Annual average cost per capita^c^	$0.0011	$0.0015	$0.0004
Average annual cost per wheat flour consumer^d^	$0.0025	$0.0035	$0.0010
Cost per kg of premix	$8.46	$7.86	−$0.60^e^
Premix cost per MT of fortified wheat flour^f^	$0.26	$0.39	$0.13
Total cost per MT of fortified wheat flour^f^	$0.53	$0.73	$0.21

*Note*: Costs modelled over 10‐year time horizon (2022–2031) and reported in undiscounted 2021 US dollars.

Abbreviation: MT, metric ton.

^a^ Under the current programme scenario, industry compliance modelled as 61.5% of wheat flour fortified with iron and folic acid at 12% of the national standard. National standard: 60 mg/kg iron and 2.5 mg/kg folic acid.

^b^ Under the expanded current programme scenario, industry compliance modelled as 61.5% of wheat flour fortified with iron, folic acid, and zinc at 12% of the hypothetical national standard. Hypothetical national standard: 60 mg/kg iron, 2.5 mg/kg folic acid, 95 mg/kg zinc.

^c^ Burkina Faso population estimates based on World Population Prospects total population 2022–2031 (United Nations et al., 2019).

^d^ The annual average number of wheat flour consumers estimated by analysis of the 2018/2019 Enquête harmonisée sur les conditions de vie des menages (EHCVM) data to estimate the percentage of the population reporting consumption of wheat flour combined with World Population Prospects estimates of the total population of Burkina Faso over 6 months of age from 2022–2031 (United Nations et al., 2019).

^e^ Note that the cost per kg of premix is lower because, to maintain the correct ratio of micronutrient fortificant to excipient, the addition rate (the rate at which premix gets added to unfortified wheat flour) jumps from 250 g of premix per MT of wheat flour without zinc to 400 g of premix per MT of wheat flour with zinc, so the concentration of micronutrient fortificants is lower with zinc (and hence the price of the premix per kg is lower) but more premix is added per MT of wheat flour.

^f^ Average annual quantity of fortified wheat flour is 50,338 metric tons.

Improving compliance of the existing programme (including only iron and folic acid), from the current state of 61.5% of wheat flour being fortified at, on average, 12% of the national standard to 90% of wheat flour being fortified to 100% of the national standard, would cost an additional ~$3 million over 10 years, or an annual average increase of $301,411 in the total cost of the programme (Table 3). In addition to new M&E costs (Supporting Information: Table S2), the premix cost would increase from $0.26 to $2.11 per MT of fortified wheat flour. Under the improved compliance scenario, adding zinc to the national standard would cost an estimated additional $77,702 per year on average, or $0.01 per wheat flour consumer per year. The increase in cost would be driven by the increase in the price of the micronutrient premix from $2.11 to $3.14 per MT of wheat flour when the premix additionally contained zinc oxide. Correspondingly, the relative contribution of the premix cost to the total cost would increase from ~48% under the improved compliance scenario with just iron and folic acid to 58% with the addition of zinc (Supporting Information: Table S2).

**Table 3:** Estimated cost of improving the performance of the current wheat flour fortification programme in Burkina Faso and the cost of expanding the improved programme to include zinc.

	Current programme^a^ (2021 US dollars)	Current programme with improved compliance^b^ (2021 US dollars)	Incremental cost of improving compliance (2021 US dollars)	Expanded programme with improved compliance^c^ (2021 US dollars)	Incremental cost of adding zinc with improved compliance (2021 US dollars)
Total 10‐year cost	$266,010	$3,280,115	$3,014,105	$4,057,132	$777,016
Annual average cost	$26,601	$328,012	$301,411	$405,713	$77,702
Annual average cost per capita^d^	$0.0011	$0.01	$0.01	$0.02	$0.003
Average annual cost per wheat flour consumer^e^	$0.0025	$0.03	$0.03	$0.04	$0.01
Cost per kg of premix	$8.46	$8.46	$0.00	$7.86	−$0.60^f^
Premix cost per MT of fortified wheat flour^g^	$0.26	$2.11	$1.85	$3.14	$1.03
Total cost per MT of fortified wheat flour^g^	$0.53	$4.72	$4.19	$5.83	$1.12

*Note*: Costs modelled over 10‐year time horizon (2022–2031) and reported in undiscounted 2021 US dollars.

Abbreviation: MT, metric ton.

^a^ Under the current programme scenario, industry compliance modelled as 61.5% of wheat flour fortified with iron and folic acid at 12% of the national standard. National standard: 60 mg/kg iron and 2.5 mg/kg folic acid.

^b^ Under the improved compliance scenario, industry compliance modelled as 90% of wheat flour fortified with iron and folic acid at 100% of the national standard. National standard: 60 mg/kg iron and 2.5 mg/kg folic acid.

^c^ Under the expanded programme with improved compliance scenario, industry compliance modelled as 90% of wheat flour fortified with iron, folic acid, and zinc at 100% of the hypothetical national standard. Hypothetical national standard: 60 mg/kg iron, 2.5 mg/kg folic acid, 95 mg/kg zinc.

^d^ Burkina Faso population estimates based on World Population Prospects total population 2022–2031 (United Nations et al., 2019).

^e^ The annual average number of wheat flour consumers estimated by analysis of the 2018/2019 Enquête harmonisée sur les conditions de vie des menages (EHCVM) data to estimate the percentage of the population reporting consumption of wheat flour combined with World Population Prospects estimates of the total population of Burkina Faso over 6 months of age from 2022–2031 (United Nations et al., 2019).

^f^ Note that the cost per kg of premix is lower because, to maintain the correct ratio of micronutrient fortificant to excipient, the addition rate (the rate at which premix gets added to unfortified wheat flour) jumps from 250 g of premix per MT of wheat flour without zinc to 400 g of premix per MT of wheat flour with zinc, so the concentration of micronutrient fortificants is lower with zinc (and hence the price of the premix per kg is lower) but more premix is added per MT of wheat flour.

^g^ Average annual quantity of fortified wheat flour is 69,565 metric tons.

### 3.3 The potential effectiveness and cost‐effectiveness of zinc‐fortified wheat flour

Based on the most recent household survey data, 44% of households in Burkina Faso report consuming any wheat flour (or product containing wheat flour) in the 7 days preceding the survey, and among those, average apparent consumption of wheat flour among consumers was 21 g/WRA/day (Table 4). Wheat flour consumption was primarily concentrated in urban areas, with 69% of urban households reporting wheat flour consumption compared with 34% in rural areas (Supporting Information: Table S3). Based on dietary intake without additional interventions to increase zinc intakes, approximately 35.5% of households had inadequate zinc density vis‐a‐vis the requirements of WRA. Zinc inadequacy was higher among rural households compared with urban households (38.2% vs. 29%). At a national level, expanding the country's wheat flour fortification standard to include zinc could reduce the prevalence of inadequate zinc density to 35.1% under the current programme, and to 31.9% assuming improved compliance with the standards (or, correspondingly, achieving effective coverage of 0.4% and 3.6%, respectively). Given wheat flour consumption patterns, the majority of these nutrition benefits would be concentrated in urban areas (Supporting Information: Table S3).

**Table 4:** Effectiveness of expanding Burkina Faso's wheat flour fortification programme to include zinc.

		Wheat flour reach^a^	Average apparent consumption of wheat flour among consumers (g/WRA/day)	Adjusted^b^ zinc density of the diet without fortification (per 1000 kcal)	Adjusted^b^ zinc density of the diet with fortification (per 1000 kcal)	Inadequate zinc density among WRA without fortification	Inadequate zinc density among WRA with fortification	Effective coverage of WRA^c^
Total	Expanded current programme^d^	44%	21	1.48	1.49	35.5%	35.1%	0.4%
	Expanded programme with improved compliance^e^	44%	21	1.48	1.56	35.5%	31.9%	3.6%
Urban	Expanded current programme^d^	69%	29	1.55	1.57	29.0%	28.0%	1.0%
	Expanded programme with improved compliance^e^	69%	29	1.55	1.74	29.0%	21.5%	7.5%
Rural	Expanded current programme^d^	34%	14	1.45	1.46	38.2%	38.1%	0.1%
	Expanded programme with improved compliance^e^	34%	14	1.45	1.48	38.2%	36.3%	1.9%

Abbreviation: WRA, women of reproductive age.

^a^ Reach defined as the percentage of women of reproductive age residing in households that reported any consumption of wheat flour or wheat flour‐containing products in the 7 days preceding the survey.

^b^ Zinc density of the household diet without and with fortification adjusted based on estimated zinc absorption calculated using published algorithms for adult women (IZiNCG, 2019).

^c^ Effective coverage defined as the percentage of women of reproductive age with inadequate zinc density from dietary intake without any interventions to increase zinc intakes who achieve adequate zinc density with wheat flour fortification with zinc.

^d^ Under the expanded current programme scenario, industry compliance modelled as 61.5% of wheat flour fortified with iron, folic acid, and zinc at 12% of the hypothetical national standard. Hypothetical national standard: 60 mg/kg iron, 2.5 mg/kg folic acid, 95 mg/kg zinc.

^e^ Under the expanded programme with improved compliance, industry compliance modelled as 90% of wheat flour fortified with iron, folic acid, and zinc at 100% of the hypothetical national standard. Hypothetical national standard: 60 mg/kg iron, 2.5 mg/kg folic acid, 95 mg/kg zinc.

Based on the total 10‐year incremental cost of expanding Burkina Faso's wheat flour fortification to include zinc and the total number of WRA effectively covered over the same time horizon, the incremental cost per WRA effectively covered would be ~$0.54 under the current programme scenario (Table 5). If compliance with the national standard improved, both the incremental cost and the number of WRA effectively covered would be higher, and the cost‐effectiveness, or incremental cost per WRA effectively covered over the 10‐year time horizon, would be $0.45.

**Table 5:** Incremental cost‐effectiveness of expanding Burkina Faso's wheat flour fortification programme to include zinc.

	Expanded current programme^a^	Expanded programme with improved compliance^b^
Total 10‐year incremental cost (2021 US dollars)	$103,470	$777,016
Number of WRA effectively covered (2022–2031)^c^	192,624	1,733,619
Incremental cost per WRA effectively covered	$0.54	$0.45

*Note*: Costs reported in undiscounted 2021 US dollars.

Abbreviation: WRA, women of reproductive age.

^a^ Under the expanded current programme scenario, industry compliance modelled as 61.5% of wheat flour fortified with iron, folic acid, and zinc at 12% of the hypothetical national standard. Hypothetical national standard: 60 mg/kg iron, 2.5 mg/kg folic acid, 95 mg/kg zinc.

^b^ Under the expanded programme with improved compliance, industry compliance modelled as 90% of wheat flour fortified with iron, folic acid, and zinc at 100% of the hypothetical national standard. Hypothetical national standard: 60 mg/kg iron, 2.5 mg/kg folic acid, 95 mg/kg zinc.

^c^ Number of WRA effectively covered based on estimate of effective coverage (defined as the percent of women of reproductive age with inadequate zinc density from dietary intake without any interventions to increase zinc intakes who achieve adequate zinc density with wheat flour fortification with zinc) multiplied by World Population prospects estimates of the total population of WRA in Burkina Faso, 2024‐2031. Effective coverage is assumed to be zero in 2022 and 2023.

### 3.4 Sensitivity analysis

If the price of zinc oxide were 20% higher or 20% lower than our assumed cost of $7.00/kg, the incremental cost per WRA effectively covered under the improved compliance scenario would be $0.52 and $0.38, respectively, compared with $0.45 reported above (Supporting Information: Table S4). If the hypothetical zinc fortification level increased by 50% to 142.5 mg/kg, under the improved compliance scenario effective coverage would increase from 3.6% to 4.6% of WRA, and the 10‐year total cost of the wheat flour fortification programme would rise to ~$1.2 million. The incremental cost per WRA effectively covered at this higher fortification level would be $0.54. Note that the risk of high intakes among WRA would remain low (<0.1%) at this higher fortification level.

## 4 DISCUSSION

Zinc deficiency remains one of the most widespread deficiencies worldwide, and while the long‐term focus on promoting the consumption of diverse, micronutrient‐dense diets should remain a priority, interventions such as LSFF can help fill dietary gaps in the more immediate term. Burkina Faso currently mandates the fortification of wheat flour with iron and folic acid. Although consumption of wheat flour is primarily concentrated in urban areas and average quantities consumed are relatively low, all of the wheat flour in Burkina Faso is industrially processed, making it a more feasible vehicle for fortification than other potential food vehicles like maize flour or rice, neither of which are industrially processed in any significant quantities (Global Fortification Data Exchange, 2022).

In this study, we used activity‐based cost modelling populated with country‐specific cost parameters and the most recent household consumption and expenditure survey data to model the potential incremental cost and cost‐effectiveness of adding zinc to Burkina Faso's current wheat flour fortification programme. We found that, although the incremental cost of fortifying wheat flour with zinc would be low ($0.01 per wheat flour consumer per year), given relatively low levels of wheat flour consumption, even with improved industry compliance with the national standard, zinc fortification would be insufficient to close the gap between zinc provided through dietary sources and zinc requirements for most WRA. Specifically, we found that, under the current programme with low industry compliance, adding zinc to the standard would increase the average annual total cost of the wheat flour fortification programme by only $10,350. However, this would only effectively cover <1% of WRA at an incremental cost of $0.54 per WRA effectively covered. We estimated that improving and sustaining improved compliance would add ~$300,000 annually, on average, to the total cost of the wheat flour fortification programme. In the context of improved compliance, adding zinc to the standard would increase the cost of the wheat flour fortification programme by an additional ~$78,000 per year, on average. More WRA (3.6%) would move from inadequate to adequate zinc density of the diet, and at an incremental cost per WRA effectively covered of $0.45 (ranging from $0.38 to $0.54 in our sensitivity analyses), the addition of zinc to the national standard with improved compliance would be more cost‐effective than under the current programme scenario. Although not the focus of this analysis, the predicted effective coverage of children age 6–59 months via zinc‐fortified wheat flour was similar to WRA (0.4% of children effectively covered under the current programme and 3.8% with improved compliance; Supporting Information: Table S4). Note that because our estimates of effective coverage were calculated based on only one target group (WRA), they are an underestimate of the total societal benefits of zinc fortification; the cost per beneficiary would decline if the benefits to other groups were included.

There is limited evidence on the cost‐effectiveness of zinc fortification and other intervention strategies to deliver zinc. Among the evidence with effectiveness metrics comparable with effective coverage, a modelling analysis of the potential cost‐effectiveness of fortifying maize flour with zinc in Zambia found that the incremental cost per person achieving adequate intake of zinc was $4.40 (Fiedler et al., 2013), although the cost of maize flour fortification included zinc plus eight other generally more expensive micronutrients. In another analysis, Vosti et al. (2023) estimated that the cost per child effectively covered via zinc fortification of wheat flour in Cameroon ranged from $0.44 to $0.62, depending on the assumed level of compliance with the national standard, which is similar to our findings in the context of Burkina Faso. In Cameroon, however, wheat flour is widely consumed (92% of WRA) (Engle‐Stone & Brown, 2015), which has contributed to the success of the introduction of mandatory wheat flour fortification in that country, including a 1‐year‐post‐fortification reduction in the prevalence of low plasma zinc concentrations from 39% to 21% and 47% to 28% among WRA and children, respectively, in the cities of Yaoundé and Douala (Engle‐Stone et al., 2017). Zinc fortification has the potential to be similarly successful in other countries where a relatively high proportion of the population consumes larger quantities of wheat flour than in Burkina Faso, such as countries like Senegal and Yemen, both of which mandate the fortification of wheat flour with iron and folic acid and have relatively high per capita availabilities of wheat flour in their food supplies (Global Fortification Data Exchange, 2022).

Our study had several limitations. One important limitation was that our measures of effectiveness were based on household food consumption data, which are less accurate than individual dietary recall data and require assumptions about the intrahousehold distribution of food (Adams, Vosti, et al., 2022). Although estimates of average apparent intake of energy and zinc among WRA were plausible (2135 kcal/day and 11.4 mg zinc/day), it is still likely that the accuracy of our estimates of zinc inadequacy and the impact of fortification were affected, to some degree, by measurement error in the underlying data. We assessed zinc adequacy based on the zinc density of the household diet to try and account for some of the measurement error inherent in household food consumption data, but these energy‐adjusted measures do not help correct systematic under or over‐reporting of foods that may vary in nutrient contents from the typical household diet, such as foods consumed away from home or foods not shared among household members. Related, because food consumed away from home was not adequately captured in the data, if food items containing wheat flour are commonly consumed outside the home, our estimates of the impact of wheat flour fortification may be low. Also, because breastfeeding status was not collected as part of the household survey, we were unable to account for zinc requirements during lactation, which are higher than during pregnancy. As a result, our estimates of the prevalence of zinc inadequacy among all WRA are likely underestimated, and our estimates of the impact of wheat flour fortification may be overestimated. As with most dietary modelling studies, it is also possible that the nutrient composition values we used (primarily from the 2019 West African FCT) may not be accurate for Burkina Faso. Finally, we were unable to model potential impacts on plasma/serum zinc concentrations, cases of diarrhoea averted, or other health outcomes. However, given low effective coverage of modelled programmes, their effects on these health outcomes would likely also be low.

Our study also had several strengths. First, we developed comprehensive, activity‐based cost models that allowed for comparison of the absolute and relative contributions of different cost components to the total cost of wheat flour fortification. Another strength was the use of those cost models, along with estimates of effectiveness, to predict cost‐effectiveness based on current compliance with the national standards, but also based on a scenario of improved compliance.

For countries with existing fortification standards for one or more food vehicles, reviewing those standards in light of new, updated, or more comprehensive evidence on micronutrient inadequacies, deficiencies, and/or intervention costs of programmes to address them may reveal opportunities for improving effectiveness and/or cost‐effectiveness of the programme by modifying fortification levels or changing the collection of micronutrients delivered via fortified foods. For example, in an effort to reduce neural tube defects, Chile expanded its wheat flour fortification standard in the Year 2000 to include folic acid (Hertrampf & Cortés, 2008). Pakistan also formally implemented a revision to its refined oil fortification standard in 2018 to require the addition of vitamin D alongside vitamin A (e‐Pact, 2019). In the case of Burkina Faso, given current wheat flour consumption patterns in which less than half of the population regularly consumes wheat flour and consumption of wheat flour among consumers is low (21 g per capita per day), considering the addition of zinc to the country's wheat flour fortification standard, even under a scenario of improved compliance, is not enough to close the nutrient gap for most people with inadequate intake. Nevertheless, adding zinc to the wheat flour fortification standard will increase zinc intake among wheat flour consumers and could be an important component of a comprehensive strategy in Burkina Faso to improve zinc adequacy via the fortification of multiple vehicles, for example, fortifying salt or bouillon with zinc in addition to wheat flour (Matthias et al., 2022; McDonald et al., 2022) and/or the release and promotion of biofortified crops such as zinc maize or zinc sorghum, both of which are categorized as high priority for Burkina Faso according to the Harvest Plus Biofortification Priority Index (HarvestPlus, 2023). Additional research to identify an economically optimal collection of effective zinc intervention programmes is warranted.

## AUTHOR CONTRIBUTIONS

Mari S. Manger, Christine M. McDonald, and Stephen A. Vosti conceptualized the study. Michael Jarvis conducted the cost analysis, with support from Katherine P. Adams and Stephen A. Vosti. Katherine P. Adams conducted the effectiveness modelling. Ann Tarini, Jérome W. Somé, and Hervé Somda contributed essential inputs into the cost and effectiveness modelling. Katherine P. Adams wrote the paper. All authors contributed to the interpretation and revisions of the manuscript, and read and approved the final manuscript.

## CONFLICT OF INTEREST STATEMENT

The authors declare no conflict of interest.

## Supporting information

Supporting information.

## Data Availability

Activity‐level cost estimates are available in Supporting Information: Tables S1 and S2. The household survey data that underlie the effectiveness estimates are publicly available for download via the World Bank Microdata Repository (https://microdata.worldbank.org/index.php/catalog/4290). See Supporting Information: Table S3 for food matches and nutrient composition information used in the analysis. While the Excel‐based cost models and Stata code used to clean, process, and analyze the household survey data are not publicly available because they are actively being used for research and policy engagement in Burkina Faso, they are available on request from the corresponding author.
