# Supplementary material for: Estimating the cost and cost‐effectiveness of adding zinc to, and improving the performance of, Burkina Faso's mandatory wheat flour fortification programme
Source: Matern Child Nutr. 2023 Apr 6;19(3):e13515. doi: 10.1111/mcn.13515 (PMC10262896; doi:10.1111/mcn.13515)
Supplement: Supplementary file 1 — Supporting information. [file MCN-19-e13515-s001.docx]

Supplemental Table S1. Activity-specific and total estimated cost of the current wheat flour fortification program in Burkina Faso and the cost of expanding the program to include zinc

|  |  | Current program^1^ | | Expanded current program^2^ | |
| --- | --- | --- | --- | --- | --- |
|  |  | Average annual cost (2021 USD) | Percent of total cost | Average annual cost (2021 USD) | Percent of total cost |
| Start-up/scale-up costs | | | | | |
|  | Mill fortification equipment (feeder/dosifier), annualized cost^3^ | 0 | 0.0% | 0 | 0.0% |
|  | Mill QA/QC equipment (iCheck portable photometers), annualized cost^3^ | 0 | 0.0% | 0 | 0.0% |
|  | Relabeling, annualized cost | 0 | 0.0% | 37 | 0.1% |
|  | Government planning (reformulation of standards, adoption of M&E plan), annualized cost | 0 | 0.0% | 2,000 | 5.4% |
|  | Government M&E equipment (iCheck portable photometers), annualized cost | 0 | 0.0% | 0 | 0.0% |
| Recurring costs | | | | | |
|  | Premix, including shipping and taxes | 16,110 | 60.6% | 24,301 | 65.8% |
|  | Mill fortification costs | 2,961 | 11.1% | 2,961 | 8.0% |
|  | Mill QA/QC activities | 6,644 | 25.0% | 6,644 | 18.0% |
|  | Mill internal training/retraining | 251 | 0.9% | 251 | 0.7% |
|  | Mill management, administration, and overhead | 635 | 2.4% | 753 | 2.0% |
|  | Government monitoring at mills | 0 | 0.0% | 0 | 0.0% |
|  | Government monitoring of imports | 0 | 0.0% | 0 | 0.0% |
|  | Government monitoring at markets and other retail outlets | 0 | 0.0% | 0 | 0.0% |
|  | Government household monitoring | 0 | 0.0% | 0 | 0.0% |
|  | Social marketing/advocacy | 0 | 0.0% | 0 | 0.0% |
|  | Capacity building/training for food control agency personnel/monitors/lab technicians | 0 | 0.0% | 0 | 0.0% |
|  | Government management, administration, and overhead | 0 | 0.0% | 0 | 0.0% |
| Total costs | | | | | |
|  | Mill-related annual average total cost | 26,601 | 100% | 34,948 | 95% |
|  | Government-related annual average total cost | 0 | 0% | 2,000 | 5% |
|  | Total annual average cost | 26,601 | 100% | 36,948 | 100% |

Costs modeled over 10-year time horizon (2022-2031) and reported in undiscounted 2021 US dollars.

^1^Under the current program scenario, industry compliance modeled as 61.5% of wheat flour fortified with iron and folic acid at 12% of the national standard. National standard: 60 mg/kg iron and 2.5 mg/kg folic acid.

^2^Under the expanded current program scenario, industry compliance modeled as 61.5% of wheat flour fortified with iron, folic acid, and zinc at 12% of the hypothetical national standard. Hypothetical national standard: 60 mg/kg iron, 2.5 mg/kg folic acid, 95 mg/kg zinc.

^3^Mill equipment costs include a 13% import duty and 18% value added tax.

Supplemental Table S2. Activity-specific and total estimated cost of improving the performance of the current wheat flour fortification program in Burkina Faso and the cost of expanding the improved program to include zinc

|  |  | Improved compliance^1^ | | Expanded program with improved compliance^2^ | |
| --- | --- | --- | --- | --- | --- |
|  |  | Average annual cost (2021 USD) | Percent of total cost | Average annual cost (2021 USD) | Percent of total cost |
| Start-up/scale-up costs | | | | | |
|  | Mill fortification equipment (feeder/dosifier), annualized cost^3^ | 1,617 | 0.5% | 1,617 | 0.4% |
|  | Mill QA/QC equipment (iCheck portable photometers), annualized cost^3^ | 541 | 0.2% | 541 | 0.1% |
|  | Relabeling, annualized cost | 37 | 0.0% | 75 | 0.0% |
|  | Government planning (reformulation of standards, adoption of M&E plan), annualized cost | 2,000 | 0.6% | 4,000 | 1.0% |
|  | Government M&E equipment (iCheck portable photometers), annualized cost | 8,468 | 2.6% | 8,468 | 2.1% |
| Recurring costs | | | | | |
|  | Premix, including shipping and taxes | 157,745 | 48.1% | 233,410 | 57.5% |
|  | Mill fortification costs | 4,848 | 1.5% | 4,848 | 1.2% |
|  | Mill QA/QC activities | 20,051 | 6.1% | 20,051 | 4.9% |
|  | Mill internal training/retraining | 1,004 | 0.3% | 1,004 | 0.2% |
|  | Mill management, administration, and overhead | 6,221 | 1.9% | 6,221 | 1.5% |
|  | Government monitoring at mills | 2,794 | 0.9% | 2,794 | 0.7% |
|  | Government monitoring of imports | 37,607 | 11.5% | 37,607 | 9.3% |
|  | Government monitoring at markets and other retail outlets | 3,273 | 1.0% | 3,273 | 0.8% |
|  | Government household monitoring | 9,000 | 2.7% | 9,000 | 2.2% |
|  | Social marketing/advocacy | 13,074 | 4.0% | 13,074 | 3.2% |
|  | Capacity building/training for food control agency personnel/monitors/lab technicians | 30,019 | 9.2% | 30,019 | 7.4% |
|  | Government management, administration, and overhead | 29,713 | 9.1% | 29,713 | 7.3% |
| Total costs | | | | | |
|  | Mill-related annual average total cost | 192,064 | 59% | 267,766 | 66% |
|  | Government-related annual average total cost | 135,947 | 41% | 137,947 | 34% |
|  | Total annual average cost | 328,012 | 100% | 405,713 | 100% |

Costs modeled over 10-year time horizon (2022-2031) and reported in undiscounted 2021 US dollars.

^1^Under the improved compliance scenario, industry compliance modeled as 90% of wheat flour fortified with iron and folic acid at 100% of the national standard. National standard: 60 mg/kg iron and 2.5 mg/kg folic acid.

^2^Under the expanded program with improved compliance scenario, industry compliance modeled as 90% of wheat flour fortified with iron, folic acid, and zinc at 100% of the hypothetical national standard. Hypothetical national standard: 60 mg/kg iron, 2.5 mg/kg folic acid, 95 mg/kg zinc.

^3^Mill equipment costs include a 13% import duty and 18% value added tax.

Supplemental Table S3. Food matches and nutrient composition of foods in the 2018/2018 Enquête Harmonisée sur les Conditions de Vie des Ménages (EHCVM) survey

| **s07bq01** | **Food in 2018 EHCVM** | **Food in 2018 EHCVM (English)** | **Nutrient composition source** | **Nutrient composition ID** | **Food name in food composition table** | **Energy  (kcal/100g)** | **Zinc  (mg/100g)** | **Phytate  (mg/100g)** |
| --- | --- | --- | --- | --- | --- | --- | --- | --- |
| 1 | Riz local (Bagré Sourou et Bama) | Local rice (Bagré Sourou and Bama) | 2019 WAFCT | 01_038 | Rice, white, boiled* (without salt), drained | 115 | 0.5 | 86.0 |
| 2 | Autre riz local (riz pluvial) | Other local rice (rainfed rice) | 2019 WAFCT | 01_038 | Rice, white, boiled* (without salt), drained | 115 | 0.5 | 86.0 |
| 3 | Riz importé long grain | Imported long grain rice | NDSR | 27418 | grains, rice, Basmati, cooked in unsalted water | 115 | 0.5 | 17.0 |
| 4 | Autre riz importé (brisure, etc.) | Other imported rice (broken, etc.) | 2019 WAFCT | 01_038 | Rice, white, boiled* (without salt), drained | 115 | 0.5 | 86.0 |
| 5 | Maïs en épi | Corn on the cob | 2019 WAFCT | 01_062 | Maize, combined varieties, whole kernel (Benin), boiled* (without salt), drained | 108 | 0.7 | 106.0 |
| 6 | Maïs en grain | Corn kernels | 2019 WAFCT | 01_062 | Maize, combined varieties, whole kernel (Benin), boiled* (without salt), drained | 108 | 0.7 | 106.0 |
| 7 | Petit mil | Millet | 2019 WAFCT | 01_064 | Pearl millet, combined varieties, whole grains (Burkina Faso), boiled* (without salt), drained | 157 | 1.0 | 189.0 |
| 8 | Sorgho | Sorghum | 2019 WAFCT | 01_042 | Sorghum, whole grains, boiled* (without salt), drained | 144 | 0.8 | 87.0 |
| 9 | Blé | wheat | 2019 WAFCT | 01_141 | Wheat, whole grains, boiled* (without salt), drained | 185 | 1.1 | 71.0 |
| 10 | Fonio | Fonio | 2019 WAFCT | 01_003 | Fonio, white, whole grains, boiled* (without salt), drained | 139 | 1.1 | 93.5 |
| 11 | Autres céréales | Other cereals | Average of sorghum and maize |  |  | 126 | 0.7 | 96.5 |
|  |  |  | 2019 WAFCT | 01_054 | Maize, yellow, meal, whole grains, unfortified | 350 | 2.0 | 630.0 |
|  |  |  | 2019 WAFCT | 01_058 | Maize, white, refined flour (special), unfortified | 350 | 1.5 | 576.1 |
| 12 | Farine de maïs | Corn flour | Weighted average of yellow corn meal (25%) and white corn flour (75%) |  |  | 350 | 1.6 | 589.6 |
| 13 | Farine de mil | Millet flour | 2019 WAFCT | 01_063 | Pearl millet, flour (bran removed) | 367 | 2.4 | 225.0 |
| 14 | Farine de blé local ou importé | Local or imported wheat flour | 2019 WAFCT | 01_043 | Wheat flour, white, unfortified | 352 | 0.5 | 123.0 |
|  |  |  | 2019 WAFCT | 01_072 | Sorghum, flour, degermed | 351 | 1.8 | 528.0 |
| 15 | Autres farines de céréales | Other cereal flours | Average of sorghum flour and maize flour |  |  | 351 | 1.7 | 558.8 |
| 16 | Pâtes alimentaires | Pasta | NDSR | 8531 | grains, pasta or noodles, spaghetti noodles, white, cooked in salted water | 158 | 0.5 | 147.0 |
|  |  |  | 2019 WAFCT | 01_048 | Bread, wheat, wholemeal, unfortified | 232 | 1.6 | 93.0 |
|  |  |  | 2019 WAFCT | 01_046 | Bread, wheat, white, unfortified | 249 | 1.0 | 93.0 |
| 17 | Pain moderne | Modern bread | Weighted average white bread (95%) and whole wheat bread (5%) |  |  | 248 | 1.0 | 93.0 |
| 18 | Pain traditionnel | Traditional bread | 2019 WAFCT | 01_046 | Bread, wheat, white, unfortified | 249 | 1.0 | 93.0 |
| 19 | Croissants | Croissants | 2019 WAFCT | 01_189 | Croissant, plain, unfortified | 373 | 0.8 | 60.4 |
| 20 | Biscuits | Biscuits | 2019 WAFCT | 01_188 | Biscuit, sweet, plain, unfortified | 479 | 0.5 | 54.9 |
| 21 | Gâteaux | Cakes | 2019 WAFCT | 01_187 | Cake, plain, unfortified | 363 | 0.4 | 24.0 |
| 22 | Beignets, galettes | Donuts, pancakes | 2019 Malawi FCT | MW01_0014 | Doughnuts (Mandasi) | 211 | 0.3 | 0.0 |
| 23 | Viande de bœuf | Beef | 2019 WAFCT | 07_086 | Beef meat, moderately fat, ca. 20% fat, stewed* (as part of a recipe) | 336 | 4.8 | 0.0 |
| 24 | Viande de chameau | Camel meat | 2019 WAFCT | 07_095 | Camel meat, stewed* (as part of a recipe) | 210 | 5.0 | 0.0 |
| 25 | Viande de mouton | Mutton | 2019 WAFCT | 07_125 | Lamb/mutton meat, moderately fat, ca. 20% fat, stewed* (as part of a recipe) | 405 | 3.7 | 0.0 |
| 26 | Viande de chèvre | Goat meat | 2019 WAFCT | 07_112 | Goat meat, moderately fat, ca. 10% fat, stewed* (as part of a recipe) | 281 | 5.9 | 0.0 |
|  |  |  | 2019 WAFCT | 07_092 | Beef tripe, stewed* (as part of a recipe) | 118 | 1.6 | 0.0 |
|  |  |  | 2019 WAFCT | 07_042 | Chicken liver, stewed* (as part of a recipe) | 152 | 4.2 | 0.0 |
|  |  |  | 2019 WAFCT | 07_089 | Beef liver, stewed* (as part of a recipe) | 171 | 4.8 | 0.0 |
|  |  |  | 2019 WAFCT | 07_020 | Beef kidney, stewed* (as part of a recipe) | 156 | 4.2 | 0.0 |
|  |  |  | 2019 WAFCt | 07_132 | Lamb liver, stewed* (as part of a recipe) | 220 | 4.7 | 0.0 |
|  |  |  | NDSR | 19280 | game, goat, liver | 191 | 5.3 | 0.0 |
| 27 | Abats et tripes (foie, rognon, etc.) | Offal and tripe (liver, kidney, etc.) | Weighted average tripe (12%), beef liver (12%), beef kidney (12%), lamb/mutton liver (27%), goat liver (23%), chicken liver (15%) |  |  | 178 | 4.3 | 0.0 |
| 28 | Viande de porc | Pork meat | 2019 WAFCT | 07_122 | Pork meat, moderately fat, ca. 20% fat, stewed* (as part of a recipe) | 425 | 3.2 | 0.0 |
| 29 | Poulet sur pied | Chicken on foot | NDSR | 12941 | poultry, chicken, whole chicken, skin eaten | 239 | 1.9 | 0.0 |
|  |  |  | 2019 WAFCT | 07_103 | Chicken, dark meat with skin, stewed* (as part of a recipe) | 331 | 2.3 | 0.0 |
|  |  |  | 2019 WAFCT | 07_104 | Chicken, light meat with skin, stewed* (as part of a recipe) | 236 | 1.0 | 0.0 |
| 30 | Viande de poulet | Chicken meat | Average of light and dark meat chicken |  |  | 284 | 1.6 | 0.0 |
|  |  |  | NDSR | 6592 | poultry, turkey, breast, unprocessed, skin eaten | 184 | 1.0 | 0.0 |
|  |  |  | NDSR | 6616 | poultry, turkey, dark meat - unknown part, skin eaten | 206 | 3.4 | 0.0 |
|  |  |  | NDSR | 6315 | poultry, duck, domestic, whole, skin eaten | 204 | 2.3 | 0.0 |
|  |  |  | 2019 WAFCT | 07_118 | Guinea fowl meat, stewed* (as part of a recipe) | 152 | 2.0 | 0.0 |
| 31 | Viande d'autres volailles domestiques | Meat of other domestic poultry | Weighted average of guinea fowl (90%), duck (5%), and light (2.5%) and dark (2.5%) meat turkey |  |  | 157 | 2.0 | 0.0 |
|  |  |  | 2019 WAFCT | 07_063 | Sausage, wiener (beef, pork, chicken) canned | 234 | 1.6 | 0.0 |
|  |  |  | 2019 WAFCT | 07_025 | Beef, corned beef, canned | 208 | 2.1 | 0.0 |
| 32 | Charcuterie (jambon, saucisson), conserves de viandes | Charcuterie (ham, sausage), canned meats | Average of canned sausage and canned beef |  |  | 221 | 1.8 | 0.0 |
| 33 | Gibiers | Game | 2019 WAFCT | 07_027 | Game meat, dried, salted | 330 | 6.1 | 0.0 |
|  |  |  | NDSR | 10791 | game, horsemeat | 175 | 3.8 | 0.0 |
|  |  |  | https://www.sciencedirect.com/science/article/pii/S0309174008001745?via%3Dihub#tbl3 |  | Skinned donkey meat (raw) | 116 | 3.7 |  |
|  |  |  | https://en.wikipedia.org/wiki/Dog_meat |  |  | 262 |  |  |
| 34 | Autres viandes n.d.a. | Other meat n.e.s. | Weighted average doneky (45%), dog (45%), and horse (10%) |  |  | 188 | 2.0 | 0.0 |
| 35 | Poisson frais carpe | Fresh carp fish | 2019 WAFCT | 09_023 | Carp, fillet, grilled* (without salt or fat) | 142 | 1.5 | 0.0 |
| 36 | Poisson frais chinchard | Fresh horse mackerel fish | 2019 WAFCT | 09_072 | Atlantic horse mackerel, wild, fillet without skin, boiled* (as part of a recipe) | 176 | 0.6 | 0.0 |
| 37 | Poisson frais maquereau | Fresh mackerel fish | 2019 WAFCT | 09_069 | Mackerel, fillet, boiled* (as part of a recipe) | 171 | 1.1 | 0.0 |
|  |  |  | 2019 WAFCT | 09_020 | Catfish, fillet, grilled* (without salt or fat) | 146 | 1.0 | 0.0 |
|  |  |  | 2019 WAFCT | 09_036 | Sardine, fillet, grilled* (without salt or fat) | 154 | 2.2 | 0.0 |
|  |  |  | 2019 WAFCT | 09_034 | Perch, Nile, fillet, grilled* (without salt or fat) | 134 | 0.8 | 0.0 |
| 38 | Autre poisson frais | Other fresh fish | Average of catfish, sardine, and perch |  |  | 145 | 1.3 | 0.0 |
| 39 | Poisson fumé siliure/carpe | Smoked catfish / carp fish | 2019 Malawi FCT | MW03_0023 | Fish, catfish, smoked, Clarias gariepinus, (Mlamba wowamba) | 434 | 5.8 | 0.0 |
| 40 | Autre poisson fumé | Other smoked fish | 2019 Malawi FCT | MW03_0023 | Fish, catfish, smoked, Clarias gariepinus, (Mlamba wowamba) | 434 | 5.8 | 0.0 |
| 41 | Poisson séché | Dried fish | Dried fish |  | Median values from multiple dried fish | 371 | 6.6 | 0.0 |
|  |  |  | 2019 WAFCT | 09_098 | Crab, flesh (body and claw), boiled* (as part of a recipe) | 107 | 4.9 | 0.0 |
|  |  |  | 2019 WAFCT | 09_107 | Shrimp, penaeid, flesh, boiled* (as part of a recipe) | 118 | 1.6 | 0.0 |
| 42 | Crabes, crevettes et autres fruits de mer | Crabs, shrimps and other seafood | Weighted average of shrimp (80%) and crab (20%) |  |  | 116 | 2.2 | 0.0 |
| 43 | Conserves de poisson | Canned fish | 2019 WAFCT | 09_037 | Sardine, canned in oil, drained, with bones | 239 | 1.8 | 0.0 |
|  |  |  | 2019 WAFCT | 10_001 | Milk, cow, whole, pasteurized or UHT, 3.5% fat | 64 | 0.6 | 0.0 |
|  |  |  | 2019 WAFCT | 10_029 | Milk, cow, whole, raw, 4.5% fat | 78 | 0.3 | 0.0 |
| 44 | Lait frais | Fresh milk | Average of pasteurized and raw milk |  |  | 71 | 0.5 | 0.0 |
|  |  |  | 2019 WAFCT | 10_005 | Yoghurt, plain, from whole cow’s milk | 82 | 0.4 | 0.0 |
|  |  |  | 2019 WAFCT | 10_022 | Milk, cow, whole, fermented | 62 | 0.3 | 0.0 |
| 45 | Lait caillé, yaourt | Curdled milk, yogurt | Weighted average of curdled milkd (80%) and yogurt (20%) |  |  | 78 | 0.3 | 0.0 |
| 46 | Lait concentré sucré | Sweetened condensed milk | 2019 WAFCT | 10_015 | Milk, cow, canned, condensed, sweetened | 329 | 1.0 | 0.0 |
| 47 | Lait concentré non-sucré | Unsweetened condensed milk | 2019 WAFCT | 10_016 | Milk, cow, canned, evaporated | 157 | 1.0 | 0.0 |
| 48 | Lait en poudre | Powdered milk | 2019 WAFCT | 10_002 | Milk, cow, powder, whole, unfortified | 493 | 3.3 | 0.0 |
| 49 | Fromage | Cheese | 2019 WAFCT | 10_028 | Cheese, fresh curd, from cow’s milk | 219 | 0.6 | 0.0 |
|  |  |  | 2019 WAFCT | 10_011 | Infant formula, powder, for 3 months, fortified | 519 | 3.9 | 0.0 |
|  |  |  | 2019 WAFCT | 10_012 | Infant formula, powder, for 6 months, fortified | 475 | 3.5 | 0.0 |
|  |  |  | 2019 Malawi FCT | MW07_0007 | Baby cereal, mixed cereal, 7 months, dry | 364 | 4.5 | 0.0 |
| 50 | Lait et farines pour bébé | Baby milk and flour | Average of infant formula (3 and 6 months), and mixed cereal |  |  | 453 | 4.0 | 0.0 |
|  |  |  | 2019 WAFCT | 10_018 | Milk, camel, fresh | 58 | 0.4 | 0.0 |
|  |  |  | 2019 WAFCT | 10_003 | Milk, goat, fresh | 83 | 0.4 | 0.0 |
| 51 | Autres produits laitiers | Other dairy products | Average of camel milk and goat milk |  |  | 71 | 0.4 | 0.0 |
| 52 | Œufs | Eggs | 2019 WAFCT | 08_002 | Egg, chicken, boiled* (without salt) | 150 | 1.4 | 0.0 |
| 53 | Beurre | Butter | 2019 WAFCT | 11_001 | Butter, from cow’s milk, unsalted | 743 | 0.1 | 0.0 |
| 54 | Beurre de karité | Shea Butter | 2019 WAFCT | 11_008 | Shea butter, white | 900 | 0.1 | 0.0 |
| 55 | Huile de palme rouge | Red palm oil | 2019 WAFCT | 11_004 | Palm oil, red | 898 | 0.0 | 0.0 |
| 56 | Huile d'arachide | Peanut oil | 2019 WAFCT | 11_003 | Groundnut oil, unfortified | 900 | 0.1 | 0.0 |
| 57 | Huile de coton | Cottonseed oil | 2019 WAFCT | 11_005 | Cottonseed oil, yellow brown, unfortified | 900 | 0.0 | 0.0 |
| 58 | Huile de soja | Soya oil | 2019 WAFCT | 11_009 | Soya oil, unfortified | 900 | 0.0 | 0.0 |
| 59 | Autres huiles n.d.a. (maïs, huile palmiste etc.) | Other oils n.e.s. (corn, palm kernel oil etc.) | 2019 WAFCT | 11_010 | Vegetable oil, unfortified | 900 | 0.0 | 0.0 |
| 60 | Mangue | Mango | 2019 WAFCT | 05_015 | Mango, orange flesh, ripe, raw | 70 | 0.1 | 82.0 |
| 61 | Ananas | Pineapple | 2019 WAFCT | 05_018 | Pineapple, fruit, raw | 53 | 0.2 | 9.0 |
| 62 | Orange | Orange | 2019 WAFCT | 05_016 | Orange, raw | 44 | 0.1 | 0.0 |
| 63 | Banane douce | Sweet banana | 2019 WAFCT | 05_028 | Banana, yellow flesh, ripe, raw | 89 | 0.3 | 14.0 |
| 64 | Citrons | Lemons | 2019 WAFCT | 05_014 | Lemon, raw | 36 | 0.1 | 0.0 |
|  |  |  | 2019 WAFCT | 05_035 | Grapefruit, white, raw | 32 | 0.1 | 0.0 |
|  |  |  | NDSR | 5386 | fruit, tangelo - fresh | 47 | 0.1 | 3.0 |
|  |  |  | NDSR | 5175 | fruit, mandarin orange, fresh | 53 | 0.1 | 50.0 |
| 65 | Autres agrumes | Other citrus | Weighted average of grapefruit (20%), tangelo (40%), and mandarin (40%) |  |  | 46 | 0.1 | 21.2 |
| 66 | Avocats | Avocados | 2019 WAFCT | 05_002 | Avocado, fruit, raw | 156 | 0.4 | 356.0 |
|  |  |  | 2019 WAFCT | 05_022 | Watermelon, fruit, raw | 25 | 0.1 | 1.0 |
|  |  |  | 2019 WAFCT | 05_039 | Melon, cantaloupe, orange flesh, raw | 38 | 0.1 | 12.0 |
|  |  |  | 2019 WAFCT | 05_038 | Melon, honeydew, pale green flesh, raw | 43 | 0.1 | 11.0 |
| 67 | Pastèque, Melon | Watermelon, Melon | Weighted average of watermelon (60%), honeydew melon (30%), and canteloupe (10%) |  |  | 32 | 0.1 | 5.1 |
| 68 | Dattes | Dates | 2019 WAFCT | 05_031 | Date, dried, raw | 282 | 0.3 | 112.0 |
| 69 | Noix de coco | Coconut | 2019 WAFCT | 06_002 | Coconut, mature kernel, fresh, raw | 383 | 0.9 | 136.0 |
| 70 | Canne à sucre | Sugar cane |  |  |  | 26 | 0.0 | 0.0 |
|  |  |  | 2019 WAFCT | 05_026 | Apple, with skin, raw | 59 | 0.1 | 1.0 |
|  |  |  | 2019 WAFCT | 05_051 | Grapes, raw | 71 | 0.1 | 1.0 |
|  |  |  | 2019 WAFCT | 05_010 | Guava, fruit, raw | 68 | 0.2 | 51.0 |
|  |  |  | 2019 WAFCT | 05_017 | Papaya, fruit, ripe, raw | 59 | 0.2 | 25.0 |
|  |  |  | 2019 WAFCT | 05_032 | Fig, raw | 74 | 0.3 | 49.0 |
|  |  |  | 2019 WAFCT | 05_044 | Shea, fruit, raw | 101 | 0.3 | 0.0 |
|  |  |  | 2019 WAFCT | 05_001 | African locust bean, fruit, raw | 285 | 0.8 |  |
| 71 | Autres fruits (pommes, raisin, etc.) | Other fruits (apples, grapes, etc.) | Average of guava, papaya (pawpaw), apples, grapes, wild figs, fruits of the African Locust bean, and shea fruit |  |  | 102 | 0.3 | 21.2 |
| 72 | Salade (laitue, choux) | Salad (lettuce, cabbage) | 2019 WAFCT | 04_040 | Lettuce, raw | 17 | 0.3 | 42.0 |
| 73 | Choux | Cabbage | 2019 WAFCT | 04_090 | Cabbage, white, boiled* (as part of a recipe) | 28 | 0.2 | 7.0 |
| 74 | Carotte | Carrot | 2019 WAFCT | 04_091 | Carrot, boiled* (as part of a recipe) | 35 | 0.3 | 11.0 |
| 75 | Haricot vert | Green bean | 2019 WAFCT | 04_088 | Bean, green, boiled* (as part of a recipe) | 43 | 0.2 | 4.0 |
| 76 | Concombre | Cucumber | 2019 WAFCT | 04_032 | Cucumber, unpeeled, raw | 12 | 0.2 | 18.0 |
|  |  |  | 2019 WAFCT | 04_102 | Eggplant, fruit, boiled* (as part of a recipe) | 39 | 0.3 | 8.0 |
|  |  |  | 2019 WAFCT | 04_115 | Pumpkin, squash, light orange flesh, boiled* (as part of a recipe) | 30 | 0.3 | 5.0 |
|  |  |  | 2019 WAFCT | 04_104 | Native eggplant, fruit, boiled* (as part of a recipe) | 40 | 1.1 | 9.0 |
| 77 | Aubergine, Courge/Courgette | Eggplant, Squash / Zucchini | Average of eggplant, pumpkin, and native eggplant |  |  | 36 | 0.6 | 7.3 |
|  |  |  | 2019 WAFCT | 04_114 | Pepper, sweet, green, fresh, boiled* (as part of a recipe) | 39 | 0.3 | 10.0 |
|  |  |  | 2019 WAFCT | 04_113 | Pepper, sweet, red, fresh, boiled* (as part of a recipe) | 56 | 0.1 | 11.0 |
| 78 | Poivron frais | Fresh bell pepper | Average of green and red pepper |  |  | 48 | 0.2 | 10.5 |
| 79 | Tomate fraîche | Fresh tomato | 2019 WAFCT | 04_124 | Tomato, red, ripe, boiled* (as part of a recipe) | 28 | 0.3 | 3.0 |
| 80 | Tomate séchée | Dried tomato | NDSR | 18442 | vegetables, tomato, sun-dried, dry pack | 258 | 2.0 | 46.7 |
| 81 | Gombo frais | Fresh okra | 2019 WAFCT | 04_111 | Okra, fruit, fresh, boiled* (as part of a recipe) | 34 | 0.6 | 10.0 |
| 82 | Gombo sec | Dry okra | 2019 WAFCT | 04_077 | Okra, fruit, dried | 245 | 4.6 | 118.0 |
| 83 | Oignon frais | Fresh onion | 2019 WAFCT | 04_112 | Onion, fresh, boiled* (as part of a recipe) | 46 | 0.4 | 5.0 |
| 84 | Ail | Garlic | 2019 WAFCT | 04_015 | Garlic, flesh, raw | 126 | 0.9 | 36.0 |
| 85 | Feuilles d'oseille | Sorrel leaves | 2019 WAFCT | 04_056 | Hibiscus, leaves, fresh, boiled* (without salt), drained | 41 | 0.5 | 42.0 |
| 86 | Feuilles de baobab | Baobab leaves | 2019 WAFCT | 04_087 | Baobab, leaves, fresh, boiled* (as part of a recipe) | 66 | 1.0 | 46.0 |
| 87 | Feuilles de haricot | Bean leaves | 2019 WAFCT | 04_098 | Cowpea, leaves, fresh, boiled* (as part of a recipe) | 39 | 0.6 |  |
|  |  |  | 2019 WAFCT | 04_105 | Eggplant, leaves, fresh, boiled* (as part of a recipe) | 51 | 0.8 | 24.0 |
|  |  |  | 2019 WAFCT | 04_101 | Moringa (drumstick), leaves, fresh, boiled* (as part of a recipe) | 86 | 1.3 | 74.0 |
|  |  |  | 2019 WAFCT | 04_116 | Pumpkin, leaves, fresh, boiled* (as part of a recipe) | 34 | 1.0 | 22.0 |
|  |  |  | 2019 WAFCT | 04_107 | Jute mallow (bush-okra), leaves, fresh, boiled* (as part of a recipe) | 53 | 0.8 | 22.0 |
|  |  |  | 2019 WAFCT | 04_118 | Spinach, leaves, fresh, boiled* (as part of a recipe) | 28 | 0.6 | 8.0 |
|  |  |  | 2019 WAFCT | 04_110 | Okra, leaves, fresh, boiled* (as part of a recipe) | 48 | 0.5 | 8.0 |
|  |  |  | 2019 WAFCT | 04_086 | Amaranth, leaves, fresh, boiled* (as part of a recipe) | 42 | 0.7 | 8.0 |
| 88 | Feuilles locales (Boulvanka) | Local leaves (Boulvanka), | Average of eggplant leaves, moringa leaves, pumpkin leaves, jute mallow leaves, spinach, okra leaves, and amaranth leaves |  |  | 49 | 0.8 | 23.7 |
|  |  |  | 2019 WAFCT | 04_092 | Cassava, leaves, fresh, boiled* (as part of a recipe) | 99 | 1.4 | 21.0 |
|  |  |  | 2019 WAFCT | 04_121 | Sweet potato, leaves, fresh, boiled* (as part of a recipe) | 54 | 0.3 |  |
| 89 | Feuilles de manioc, taro et autres | Cassava leaves, taro and others | Average of cassava leaves and sweet potato leaves |  |  | 77 | 0.8 | 21.0 |
|  |  |  | Values from INDDEX study (source Becquey) |  | Chalice Kapok flower, fresh, raw | 62 | 0.2 |  |
|  |  |  | Values from INDDEX study (source Becquey) |  | Chalice Kapok flower, dried, raw | 295 | 2.5 |  |
| 90 | Kapok (voaga) et autre légumes frais n.d.a. | Kapok (voaga) and other fresh vegetables n.e.s. | Average of fresh an dried kapok |  |  | 179 | 1.4 |  |
| 91 | Concentré de tomate | Tomato concentrate | 2019 WAFCT | 04_066 | Tomato, paste, concentrated, without salt | 80 | 0.7 |  |
| 92 | Petits pois | Peas | NDSR | 3057 | vegetables, peas, green peas, cooked from fresh | 84 | 1.2 | 80.0 |
| 93 | Petit pois secs (Voandzou) | Dry peas (Voandzou) | 2019 WAFCT | 03_001 | Bambara groundnut, dry, raw | 323 | 2.4 |  |
|  |  |  | 2019 WAFCT | 03_034 | Soya bean, not soaked, boiled* (without salt), drained | 157 | 1.8 | 162.0 |
|  |  |  | 2019 WAFCT | 03_031 | Lentil, not soaked, boiled* (without salt), drained | 124 | 1.3 | 172.0 |
| 94 | Autres légumes secs n.d.a. | Other pulses n.e.s. | Weighted average of soyabeans (80%) and lentils (20%) |  |  | 150 | 1.7 | 164.0 |
| 95 | Niébé/Haricots secs | Cowpea / Dried beans | 2019 WAFCT | 03_007 | Cowpea, not soaked, boiled* (without salt), drained | 122 | 1.2 | 236.0 |
| 96 | Arachides fraîches en coques | Fresh peanuts in shell | 2019 WAFCT | 06_010 | Groundnut, shelled, dried, raw | 574 | 2.6 | 339.0 |
| 97 | Arachides séchées en coques | Dried peanuts in shell | 2019 WAFCT | 06_010 | Groundnut, shelled, dried, raw | 574 | 2.6 | 339.0 |
| 98 | Arachides décortiquées ou pilées | Peanuts, shelled or crushed | 2019 WAFCT | 06_010 | Groundnut, shelled, dried, raw | 574 | 2.6 | 339.0 |
| 99 | Arachide grillée | Roasted peanut | NDSR | 2198 | nuts and seeds, peanuts, roasted, dry roasted, unsalted | 587 | 2.8 | 448.0 |
| 100 | Pâte d'arachide | Peanut paste | 2019 WAFCT | 06_026 | Groundnut paste, from groundnuts only | 605 | 2.6 | 339.0 |
| 101 | Sésame | Sesame | 2019 WAFCT | 06_015 | Sesame seed, whole, dried, raw | 601 | 2.1 | 898.0 |
| 102 | Noix de cajou | Cashew nut | 2019 WAFCT | 06_001 | Cashew nut, raw | 591 | 5.3 | 0.0 |
| 103 | Noix de karité | Shea nuts | 2019 WAFCT | 05_044 | Shea, fruit, raw | 101 | 0.3 | 0.0 |
| 104 | Manioc | Cassava | 2019 WAFCT | 02_003 | Cassava, tuber, white flesh, boiled* (without salt), drained | 121 | 1.6 | 0.0 |
| 105 | Igname | Yam | 2019 WAFCT | 02_020 | Yam, tuber, pale, boiled* (without salt), drained | 132 | 0.6 | 0.0 |
| 106 | Plantain | Plantain | 2019 WAFCT | 02_057 | Plantain, ripe, ivory flesh, boiled* (without salt), drained | 151 | 0.1 | 0.0 |
| 107 | Pomme de terre | Potato | 2019 WAFCT | 02_010 | Potato, boiled* (without salt), drained | 79 | 0.3 | 0.0 |
| 108 | Taro, macabo | Taro, macabo | 2019 WAFCT | 02_016 | Taro, tuber, white, boiled* (without salt), drained | 125 | 0.5 | 0.0 |
| 109 | Patate douce | Yam | 2019 WAFCT | 02_023 | Sweet potato, pale flesh (white/cream/pale yellow), boiled* (without salt), drained | 96 | 0.5 | 0.0 |
| 110 | Autres tubercules n.d.a. | Other tubers n.e.s. | Average of included tubers (excluding plantain) |  |  | 111 | 0.7 | 0.0 |
| 111 | Farines de manioc | Cassava flour | 2019 WAFCT | 02_004 | Cassava, flour, from white cassava | 341 | 0.2 | 0.0 |
| 112 | Gari, tapioca | Gari, tapioca | 2019 WAFCT | 02_039 | Cassava, grated, from fermented white cassava, toasted without oil (white gari) | 351 | 0.7 | 0.0 |
| 113 | Attiéke | Attiéke | https://www.scirp.org/journal/paperinformation.aspx?paperid=67396 |  | Attiéke | 186 | 0.4 |  |
| 114 | Sucre (poudre ou morceaux) | Sugar (powder or lumps) | 2019 WAFCT | 13_002 | Sugar, white | 400 | 0.0 | 0.0 |
| 115 | Miel | Honey | 2019 WAFCT | 13_001 | Honey | 326 | 0.2 | 0.0 |
| 116 | Chocolat à croquer, pâte à tartiner | Chewable chocolate, spread | NDSR | 103855 | nuts and seeds, filberts, spread (e.g. Nutella) | 541 | 1.1 | 129.1 |
| 117 | Caramel, bonbons, confiseries, etc. | Caramel, candy, confectionery, etc. | NDSR | 3614 | candy, other confections (non-chocolate), hard candy, regular | 394 | 0.0 | 0.0 |
| 118 | Sel | Salt | 2019 WAFCT | 13_015 | Salt | 0 | 0.1 | 0.0 |
|  |  |  | 2019 WAFCT | 13_006 | Chilli pepper, dried | 347 | 1.2 | 0.0 |
|  |  |  | 2019 WAFCT | 04_046 | Pepper, chilli, fresh, raw | 47 | 0.3 | 0.0 |
| 119 | Piment | chilli pepper |  |  | Average of fresh and dried chilli pepper | 197 | 0.8 | 0.0 |
|  |  |  | 2019 WAFCT | 13_011 | Ginger, root, dried | 329 | 4.2 | 0.0 |
|  |  |  | 2019 WAFCT | 04_082 | Ginger, root, raw | 63 | 0.4 | 0.0 |
| 120 | Gingembre | Ginger |  |  | Average of fresh and dried ginger | 196 | 2.3 | 0.0 |
| 121 | Cube alimentaire (Maggi, Jumbo, ) | Food cube (Maggi, Jumbo,) | 2019 WAFCT | 13_008 | Cube, beef, dry | 190 | 0.5 | 0.0 |
| 122 | Arôme (Maggi, Jumbo, etc.) | Aroma (Maggi, Jumbo, etc.) |  |  |  | 102 |  |  |
| 123 | Soumbala (moutarde africaine) | Soumbala (African mustard) | 2019 WAFCT | 03_042 | African locust bean, fermented (soumbala) | 375 | 4.7 | 0.0 |
| 124 | Mayonnaise | Mayonnaise | NDSR | 10982 | dressing for salads, mayonnaise or mayo type dressing, real, regular, commercial | 680 | 0.2 | 0.0 |
|  |  |  | 2019 WAFCT | 13_003 | Vinegar | 19 | 0.0 | 0.0 |
|  |  |  | 2019 WAFCT | 13_013 | Mustard, prepared, yellow | 60 | 0.6 | 0.0 |
| 125 | Vinaigre /moutarde | Vinegar / mustard |  |  | Average of vinegar and mustard | 40 | 0.3 | 0.0 |
| 126 | Autres condiments (poivre etc.) | Other condiments (pepper etc.) | 2019 WAFCT | 13_014 | Pepper, black | 276 | 1.2 | 0.0 |
| 127 | Noix de cola | Cola nuts | 2019 WAFCT | 06_019 | Cola nut, dried, raw | 282 | 2.5 | 0.0 |
|  |  |  | 2019 WAFCT | 06_018 | Cola nut, fresh, raw | 135 | 1.2 | 0.0 |
|  |  |  |  |  | Average of fresh and dried cola nuts | 209 | 1.8 | 0.0 |
| 129 | Café | Coffee | 2019 WAFCT | 12_009 | Coffee, liquid | 2 | 0.0 | 141.0 |
| 130 | Thé | Tea | 2019 WAFCT | 12_008 | Tea, infusion | 0 | 0.0 | 0.0 |
| 131 | Chocolat en poudre | Chocolate powder | NDSR | 25488 | milk, mixtures and milk drinks, cocoa or hot chocolate, dry mix - unprepared, regular - to be mixed with milk, brands, Nestle Nesquik - dry mix, chocolate flavors | 400 | 9.4 | 441.4 |
| 132 | Autres tisanes et infusions n.d.a. (quinquelibat, citronelle, etc.) | Other herbal teas and infusions n.e.s. (quinquelibat, citronella, etc.) | 2019 WAFCT | 12_008 | Tea, infusion | 0 | 0.0 | 0.0 |
| 133 | Jus de fruits (orange, bissap, etc.) | Fruit juice (orange, bissap, etc.) | 2019 WAFCT | 12_012 | Juice, canned or bottled, sweetened (e.g. apple) | 48 | 0.0 | 0.0 |
| 134 | Eau minérale filtrée | Filtered mineral water | 2019 WAFCT | 12_019 | Water, tap | 0 | 0 | 0.0 |
| 135 | Boissons gazeuses (coca, etc.) | Soft drinks (cola, etc.) | 2019 WAFCT | 12_024 | Carbonated drink (e.g. Coca-Cola, Sprite) | 40 | 0.1 | 0.0 |
| 136 | Jus en poudre | Powdered juice | NDSR | 27898 | beverages, juice or flavored drink, brand name listing, Kraft, Tang - sweetened, all flavors | 42 | 0.0 | 0.0 |
| 137 | Bières traditionnelles (dolo, vin de palme, vin de raphia, etc.) | Traditional beers (dolo, palm wine, raffia wine, etc.) | 2019 WAFCT | 12_003 | Beer, millet (ca. 3% v/v alcohol) | 43 | 0.1 | 0.0 |
| 138 | Bières industrielles | Industrial beers | 2019 WAFCT | 12_001 | Beer, European (4.6% v/v alcohol) | 41 | 0.0 | 0.0 |

Supplemental Table S4. Effectiveness of expanding Burkina Faso’s wheat flour fortification program to include zinc among children 6-59 months of age

|  |  | Wheat flour reach^3^ | Average apparent consumption of wheat flour among consumers (g/child/day) | Inadequate zinc density^4^ among children without fortification | Inadequate zinc density among children with fortification | Effective coverage of children^5^ |
| --- | --- | --- | --- | --- | --- | --- |
| Total | Expanded current program^1^ | 43% | 9 | 45.4% | 45.0% | 0.4% |
|  | Expanded program with improved compliance^2^ | 43% | 9 | 45.4% | 41.6% | 3.8% |
| Urban | Expanded current program^1^ | 70% | 13 | 50.3% | 49.2% | 1.1% |
|  | Expanded program with improved compliance^2^ | 70% | 13 | 50.3% | 39.4% | 10.9% |
| Rural | Expanded current program^1^ | 35% | 6 | 43.8% | 43.6% | 0.2% |
|  | Expanded program with improved compliance^2^ | 35% | 6 | 43.8% | 42.3% | 1.5% |

^1^Under the expanded current program scenario, industry compliance modeled as 61.5% of wheat flour fortified with iron, folic acid, and zinc at 12% of the hypothetical national standard. Hypothetical national standard: 60 mg/kg iron, 2.5 mg/kg folic acid, 95 mg/kg zinc.

^2^Under the expanded program with improved compliance, industry compliance modeled as 90% of wheat flour fortified with iron, folic acid, and zinc at 100% of the hypothetical national standard. Hypothetical national standard: 60 mg/kg iron, 2.5 mg/kg folic acid, 95 mg/kg zinc.

^3^Reach defined as the percent of children age 6-59 months of age residing in households that reported any consumption of wheat flour or wheat flour-containing products in the seven days preceding the survey.

^4^Zinc absorption among children was estimated using published algorithms (Miller, Hambidge & Krebs, 2015). We then adjusted the EARs and critical nutrient densities for zinc based on the percent absorbed zinc among children the household samples.

^5^Effective coverage defined as the percent of children 6-59 months of age with inadequate zinc density from diets alone who achieve adequate zinc density with zinc-fortified wheat flour fortification with zinc.

Supplemental Table S4. Sensitivity analysis: incremental cost-effectiveness of expanding Burkina Faso’s wheat flour fortification program to include zinc with improved compliance

|  | Primary analysis | 20% increase in the price of zinc oxide^1^ | 20% decrease in the price of zinc oxide^2^ | 50% increase in the zinc fortification level^3^ |
| --- | --- | --- | --- | --- |
| WRA effectively covered^4^ (%) | 3.6% | 3.6% | 3.6% | 4.6% |
| Total 10-year incremental cost (2021 US dollars) | $777,016 | $896,433 | $657,600 | $1,203,255 |
| Number of WRA effectively covered (2022-2031)^3^ | 1,733,619 | 1,733,619 | 1,733,619 | 2,215,179 |
| Incremental cost per WRA effectively covered | $0.45 | $0.52 | $0.38 | $0.54 |

Costs reported in undiscounted 2021 US dollars.

^1^Price of zinc oxide modeled at $8.40 per kg, compared to $7.00 per kg in the primary analysis.

^2^Price of zinc oxide modeled at $5.60 per kg, compared to $7.00 per kg in the primary analysis.

^3^Fortificaion level modeled as 142.5 mg/kg compared 95 mg/kg in the primary analysis.

^4^Number of WRA effectively covered based on estimate of effective coverage (defined as the percent of women of reproductive age with inadequate zinc density from diets alone who achieve adequate zinc density with wheat flour fortification with zinc) multiplied by World Population prospects estimates of the total population of WRA in Burkina Faso, 2024-2031. Effective coverage assumed to be zero in 2022 and 2023.

**References**

Miller L.V., Hambidge K.M. & Krebs N.F. (2015). Zinc absorption is not related to dietary phytate intake in infants and young children based on modeling combined data from multiple studies. *J Nutr,* 145, 1763-1769.
